# Supplementary material for: C-type lectin receptor CLEC4A2 promotes tissue adaptation of macrophages and protects against atherosclerosis
Source: Nat Commun. 2022 Jan 11;13:215. doi: 10.1038/s41467-021-27862-9 (PMC8752790; doi:10.1038/s41467-021-27862-9)
Supplement: Supplementary file 1 — Supplementary Information [file 41467_2021_27862_MOESM1_ESM.pdf]

C-type lectin receptor CLEC4A2 promotes tissue adaptation of macrophages and protects against atherosclerosis

Author Information

Inhye Park[1], Michael Goddard[1,5], Jennifer Cole[1,5], Natacha Zanin[1], Leo-Pekka Lyytikäinen[2], Terho Lehtimäki[2], Evangelos Andreakos[3], Marc Feldmann[1], Irina Udalova[1], Ignat Drozdov[4], Claudia Monaco[1,*]

SUPPLEMENTARY FIGURES AND TABLES

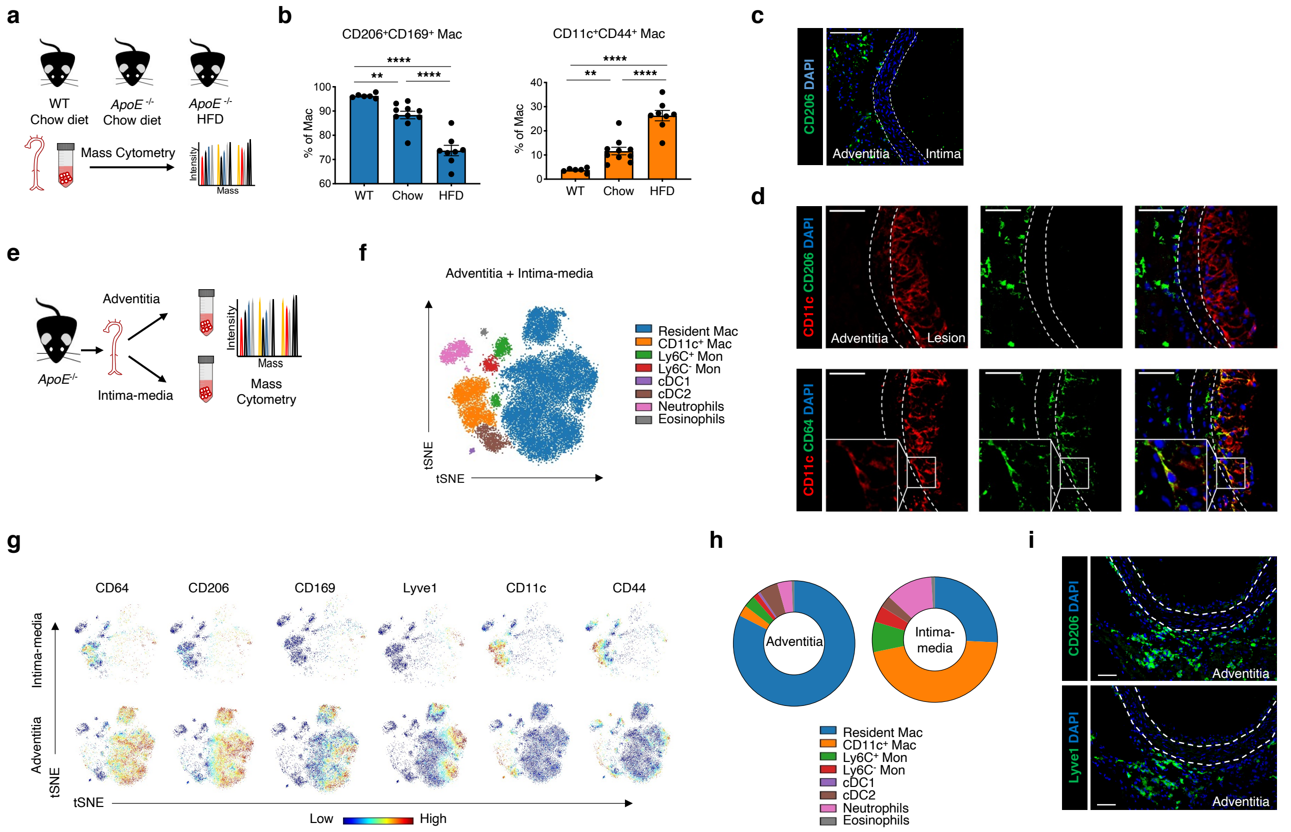

Supplementary Figure 1. Atherosclerotic aortas harbour adventitial and lesional macrophage compartments.

a, Single cells from whole aortas of C57BL/6J wild type (WT) (chow-fed), *ApoE*$^{-/-}$ (chow-fed) or *ApoE*$^{-/-}$ (high fat diet (HFD)-fed) mice were analysed by mass cytometry. **b,** The proportion of resident macrophages and CD11c^{+} macrophages in each group (mean±SEM). WT: n=6, Chow: n=10 and HFD: n=8. For each sample, cells from two aortas were pool. The data are pooled from three experiments. Ordinary one-way ANOVA with Holm-Sidak's multiple comparisons test. CD206^{+}CD169^{+} Mac: **P=0.0043, ****<0.0001. CD11c^{+} Mac: **P=0.0043, ****<0.0001. **c**, Immuno-staining of CD206 in the thoracic aorta of WT mice. (n=3 mice; Scale bar: 100μm; white dotted lines indicate elastic laminae). **d**, Immuno-staining of CD11c and CD206 or CD11c and CD64 in the aortic root of HFD-fed *ApoE*$^{-/-}$ mice (n=6 mice; Scale bar: 200μm; white dotted lines indicate elastic laminae). **e,** Single cells from separated intima-media and adventitial layers of aortas from chow-fed *ApoE*$^{-/-}$ mice were analysed by mass cytometry. **f**, 8 myeloid cell populations (CD11b$^{\text{int-high}}$) were identified in concatenated files of intima-media and adventitia by tSNE clustering based on the expression of 21 markers. (12 aortas were pooled; the data are representative of two independent experiments). **g**, tSNE images displaying the expression of CD64, CD206, CD169, Lyve1, CD11c and CD44 in each compartment. **h**, Proportion of 8 myeloid cell populations in each compartment**. i**, Immuno-staining of CD206 and Lyve1 in the aortic root of HFD-fed *ApoE*$^{-/-}$ mice (n=3 mice; Scale bar: 100μm; white dotted lines indicate elastic laminae). Source data are provided as a Source Data file.

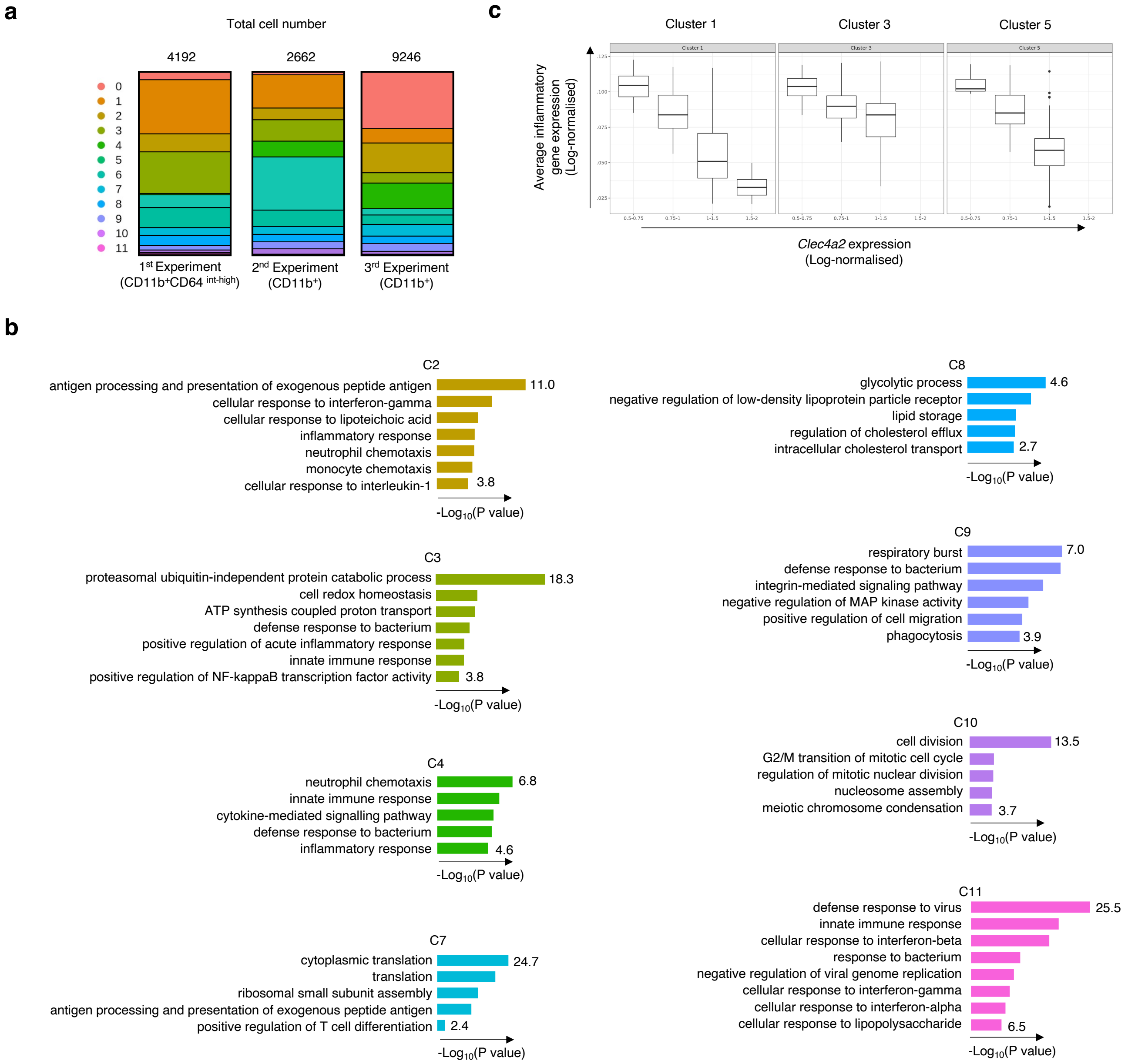

Supplementary Figure 2 (related to Figure 1).

Total cells from whole aortas of 9 *ApoE*$^{-/-}$ mice fed an HFD for 12-16 weeks were sorted as live CD45^{+}CD11b^{+}CD64$^{int\text{-}high}$ (first experiment) or live CD45^{+}CD11b^{+} (second and third experiments) populations. These cells were analysed by single cell RNA-sequencing (scRNA-seq) using the 10X genomics platform. **a,** Total cell numbers and the proportion of each cluster in three independent experiments. **b,** The Gene Ontology (GO) enrichment analysis of clusters based on differentially expressed genes in myeloid clusters. Enriched pathways are presented as -Log10 (p-values) using topGO analysis. P values were obtained using the one-sided Fisher's exact test without multiple testing correction. P values <0.05 were considered significant. **c,** Genetic association between *Clec4a2* and the average expression of 170 inflammatory genes in Cluster 1, 3 and 5. The boxes show the 25 % to 75 % percentile with median; whiskers encompass 1.5× the interquartile range, and data beyond that threshold indicated as outliers. Source data are provided as a Source Data file.

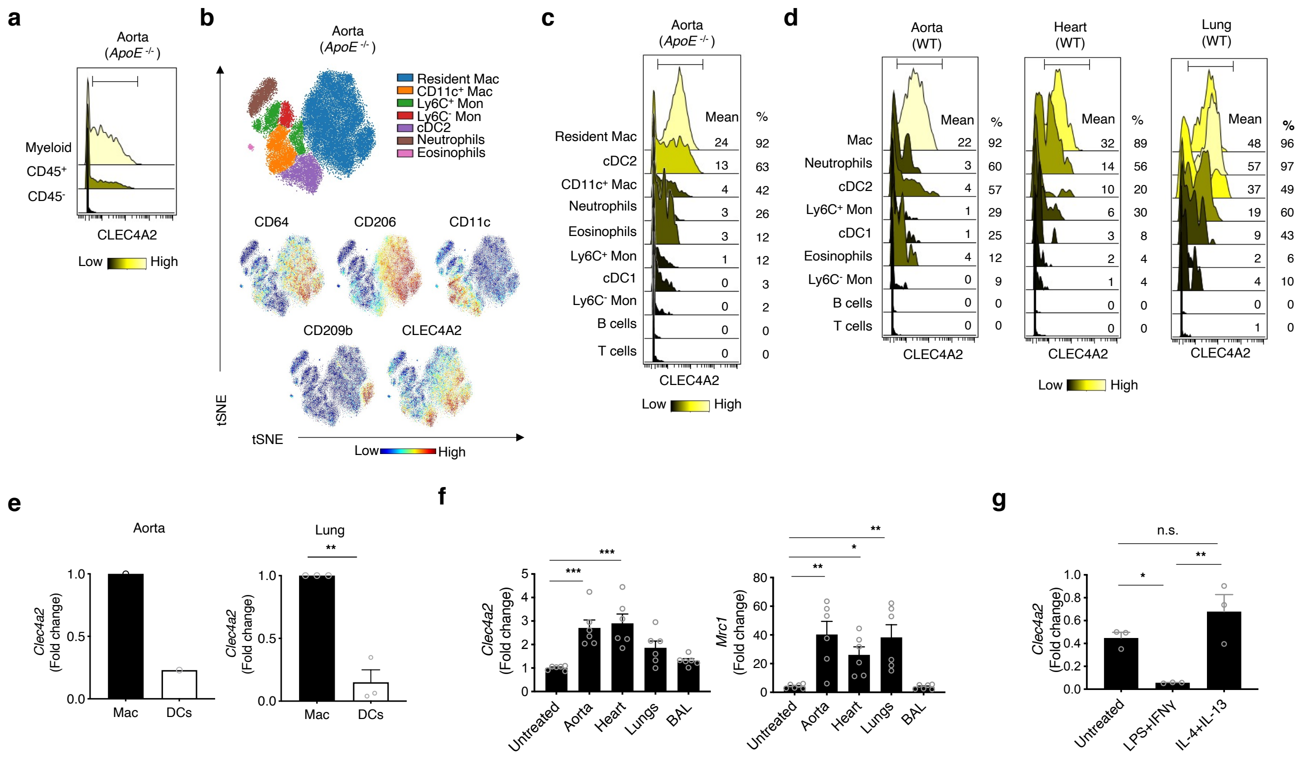

Supplementary Figure 3. CLEC4A2 expression in leucocytes from murine organs and in bone marrow-derived macrophages.

a, A representative histogram showing the mean of CLEC4A2 expression in $CD45^+CD11b^+$ cells, $CD45^+$ cells and $CD45^-$ cells from whole aortas of *ApoE*$^{-/-}$ mice fed an HFD for 12 weeks by mass cytometry. **b,** Overlaid tSNE plot of aortic $CD45^+CD11b^+$ cells from *ApoE*$^{-/-}$ mice. Myeloid populations were clustered based on the expression of 21 markers. Selected marker expression of vascular myeloid cells in the tSNE projection. **c**, A representative histogram showing the mean of CLEC4A2 expression and % of $CLEC4A2^+$ cells in 10 vascular leucocyte populations of HFD-fed *ApoE*$^{-/-}$ mice by mass cytometry (n=4 mice). **d,** Representative histograms showing the mean of CLEC4A2 expression and % of $CLEC4A2^+$ cells in 9 leucocytes from aortas, hearts and lungs of WT mice by mass cytometry. (n=4 mice). **e,** Gene expression of *Clec4a2* in sorted macrophages ($CD45^+CD11b^+CD64^+F4/80^+$) and dendritic cells (DCs; ($CD45^+CD11c^+MHCII^+CD64^-$) from the whole aorta and lungs. Aorta n=1 (9 mice pooled); Lungs n=3 mice. Two-tailed Student's t-test. **P=0.0010. **f**, Gene expression of *Clec4a2* and *Mrc1* in CSF1-cultured bone marrow-derived macrophages (BMDMs) after treating with conditioned media from aortas, hearts, lungs or broncho-alveolar lavage (BAL) for 24 hours. n=6 mice each; pooled from two independent experiments. Ordinary one-way ANOVA with Holm-Sidak's multiple comparisons test. *Clec4a2*: ***P=0.0004 (Aorta), ***P=0.0002 (Heart). *Mrc1*: **P=0.0015 (Aorta), *P=0.0383 (Heart), **P=0.0020. **g**, Gene expression of *Clec4a2* in CSF1-BMDMs in response to lipopolysaccharide (LPS) + interferon (IFN)γ or interleukin (IL)-4 + IL-13. n=3 mice. Ordinary one-way ANOVA with Holm-Sidak's multiple comparisons test. P=0.0443, **P=0.0086. not significant (n.s.). All data are presented as mean±SEM. Source data are provided as a Source Data file.

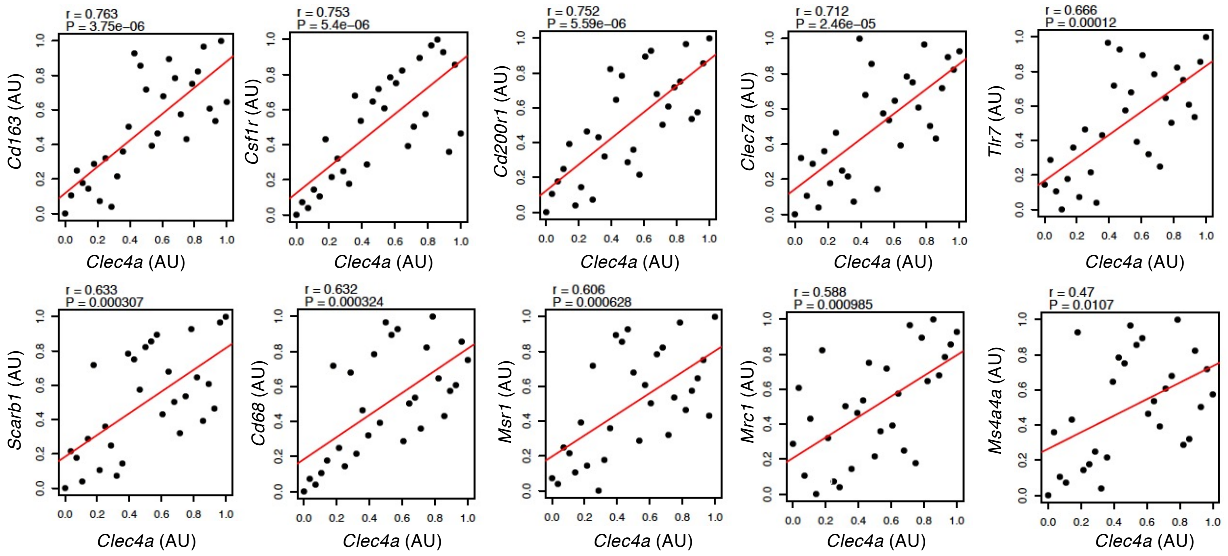

Supplementary Figure 4. Correlation of *Clec4a* expression with macrophage markers in the human atherosclerotic tissue.

Scatter plot graphs showing a positive correlation between expression of *Clec4a* and myeloid marker genes in human carotid endarterectomies (n=29) by Spearman’s rank correlation. Gene expression data were obtained from the Tampere Vascular Study. AU stands for arbitrary unit.

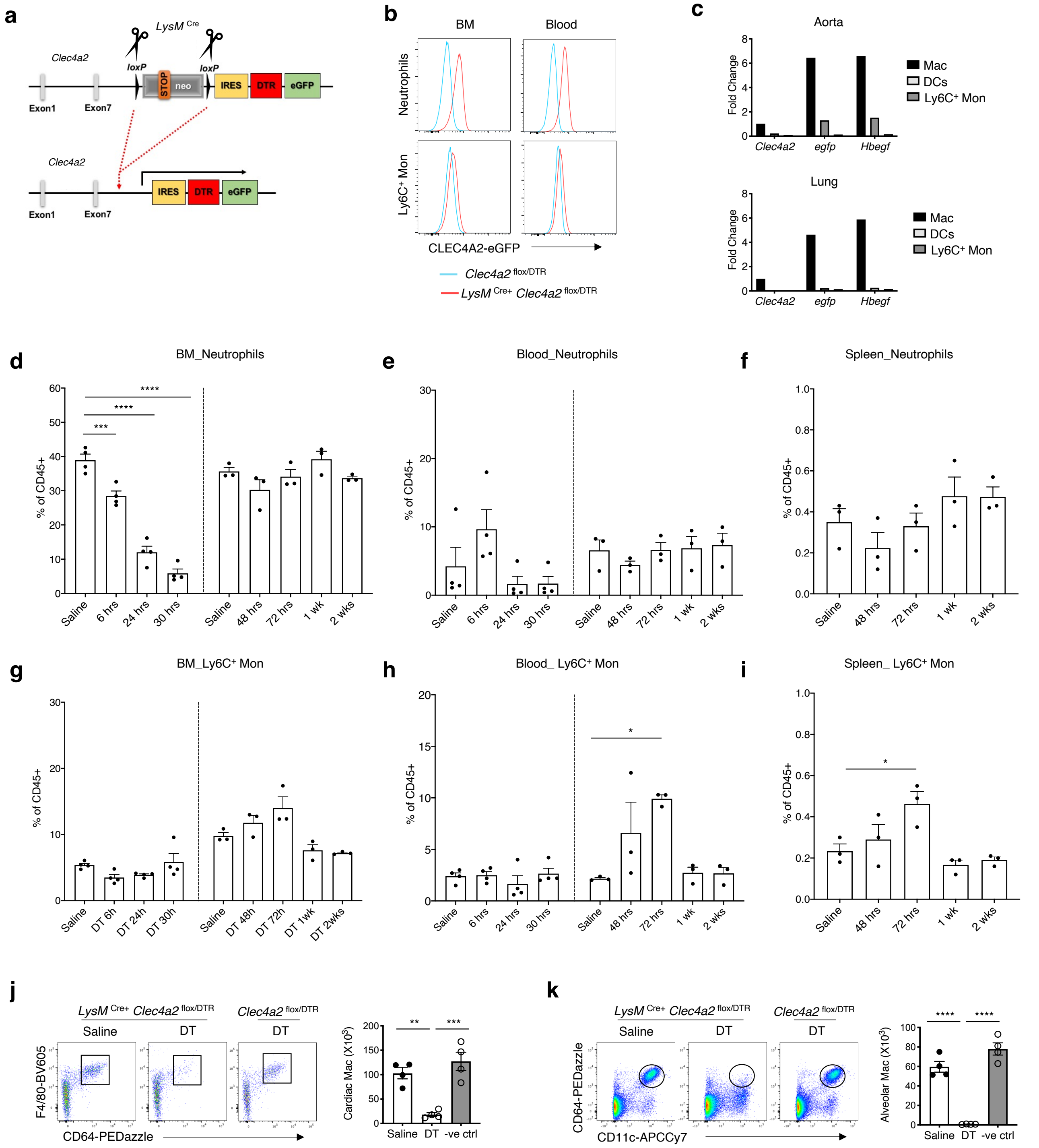

Supplementary Figure 5. (related to Figure 2).

a, The gene construct for tracing and ablating cells expressing both *LysM* and *Clec4a2*. **b,** Endogenous expression of eGFP in neutrophils and Ly6C+ monocytes in the bone marrow (BM) and blood of *Clec4a2*$^{flox/DTR}$ and *LysM*$^{Cre+}$ *Clec4a2*$^{flox/DTR}$ mice. **c,** Gene expression of *Clec4a2, egfp and Hbegf (DTR)* in sorted macrophages ($CD45^+CD11b^+CD64^+F4/80^+$), Ly6C+ monocytes ($CD45^+CD11b^+Ly6C^+CD64^-$) and DCs ($CD45^+CD11c^+MHCII^+CD64^-$) from the whole aorta and lungs of *LysM*$^{Cre+}$ *Clec4a2*$^{flox/DTR}$ mice by RT-qPCR. **d-i**, Percentages of neutrophils and Ly6C+ monocytes from the BM, blood and spleen of *LysM*$^{Cre+}$ *Clec4a2*$^{flox/DTR}$

mice at 6, 24, 30, 48, 72 hours and 1- and 2-weeks post a single diphtheria toxin (DT) injection (n=3-4 mice each; one experiment). Ordinary one-way ANOVA with Holm-Sidak's multiple comparisons test. d: ***P=0.0005, ****P<0.0001. e,f: not significant, h: **P=0.0107. i: *P=0.0225. **j,k**, Representative flow cytometry plots showing macrophage depletion and cell numbers in the heart and lungs of *LysM*$^{Cre+}$ *Clec4a2*$^{flox/DTR}$ and *Clec4a2*$^{flox/DTR}$ mice that received three intraperitoneal injections of saline or DT in a week (n=4 mice; the data are representative of three independent experiments). Ordinary one-way ANOVA with Holm-Sidak's multiple comparisons test. Cardiac Mac: **P=0.0024, ***P=0.0006. Alveolar Mac: ****P<0.0001. All data are presented as mean±SEM. Source data are provided as a Source Data file.

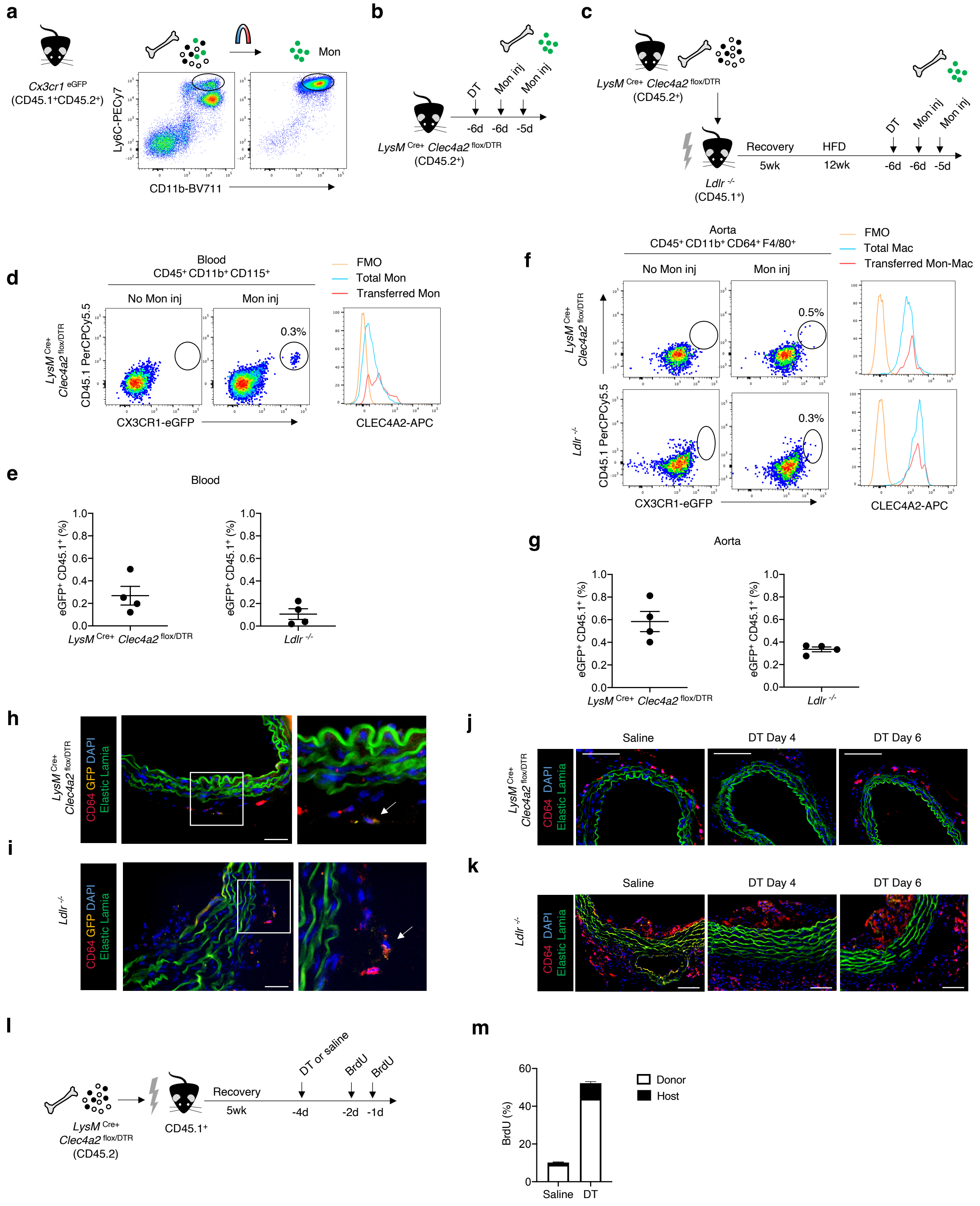

Supplementary Figure 6 (related to Figure 2). Replenishment of vascular macrophages by Ly6C+ monocytes post ablation using *LysM*Cre+ *Clec4a2*flox/DTR mice. a, Enrichment of Ly6C+ monocytes from the BM of *Cx3cr1*eGFP+ CD45.1+CD45.2+ mice using magnetic bead negative isolation. **b**, Schematic of DT-mediated macrophage ablation and adoptive transfer of monocytes in *LysM*Cre+ *Clec4a2*flox/DTR mice. **c**, Schematic of DT-mediated macrophage ablation and adoptive transfer of monocytes in atherosclerotic mice. BM cells from

LysM^{Cre+} *Clec4a2*$^{flox/DTR}$ mice were transplanted into *Ldlr*$^{-/-}$ CD45.1^{+} mice. Chimeric mice received an HFD for 12 weeks. Mice were subject to a DT injection followed by adoptive transfer of monocytes. **d**, **e**, Detection of transferred monocytes in the blood of healthy and atherosclerotic mice by flow cytometry. The mean fluorescent intensity (MFI) of CLEC4A2 expression in monocytes from the host and donor mice (n=4 mice; one experiment). FMO stands for fluorescence minus one. **f**, **g**, Detection of transferred monocytes in the whole aorta of healthy and atherosclerotic mice by flow cytometry. The MFI of CLEC4A2 expression in macrophages from the host and donor mice (n=4 mice; one experiment). **h**, **i**, Detection of transferred monocytes using immuno-staining for CD64 and GFP in the femoral artery of *LysM*$^{Cre+}$ *Clec4a2*$^{flox/DTR}$ mice and the ascending aorta of the *Ldlr*$^{-/-}$ chimeric mice (n=4 mice; Scale bar: 100μm). **j**, **k**, Immuno-staining for CD64 in the femoral artery of *LysM*$^{Cre+}$ *Clec4a2*$^{flox/DTR}$ mice and the ascending aorta of the *Ldlr*$^{-/-}$ chimeric mice, showing depletion and replenishment of adventitial macrophages (n=4 mice; Scale bar: 100μm). **l**, Schematic of macrophage depletion and proliferation assays in chimeric CD45.1^{+} mice reconstituted with the BM from *LysM*$^{Cre+}$ *Clec4a2*$^{flox/DTR}$ mice. **m**, Percentages of BrdU incorporation in vascular macrophages derived from donor (CD45.2) and host (CD45.1) origins post saline or DT treatment (n=3 mice; one experiment). All data are presented as mean±SEM. Source data are provided as a Source Data file.

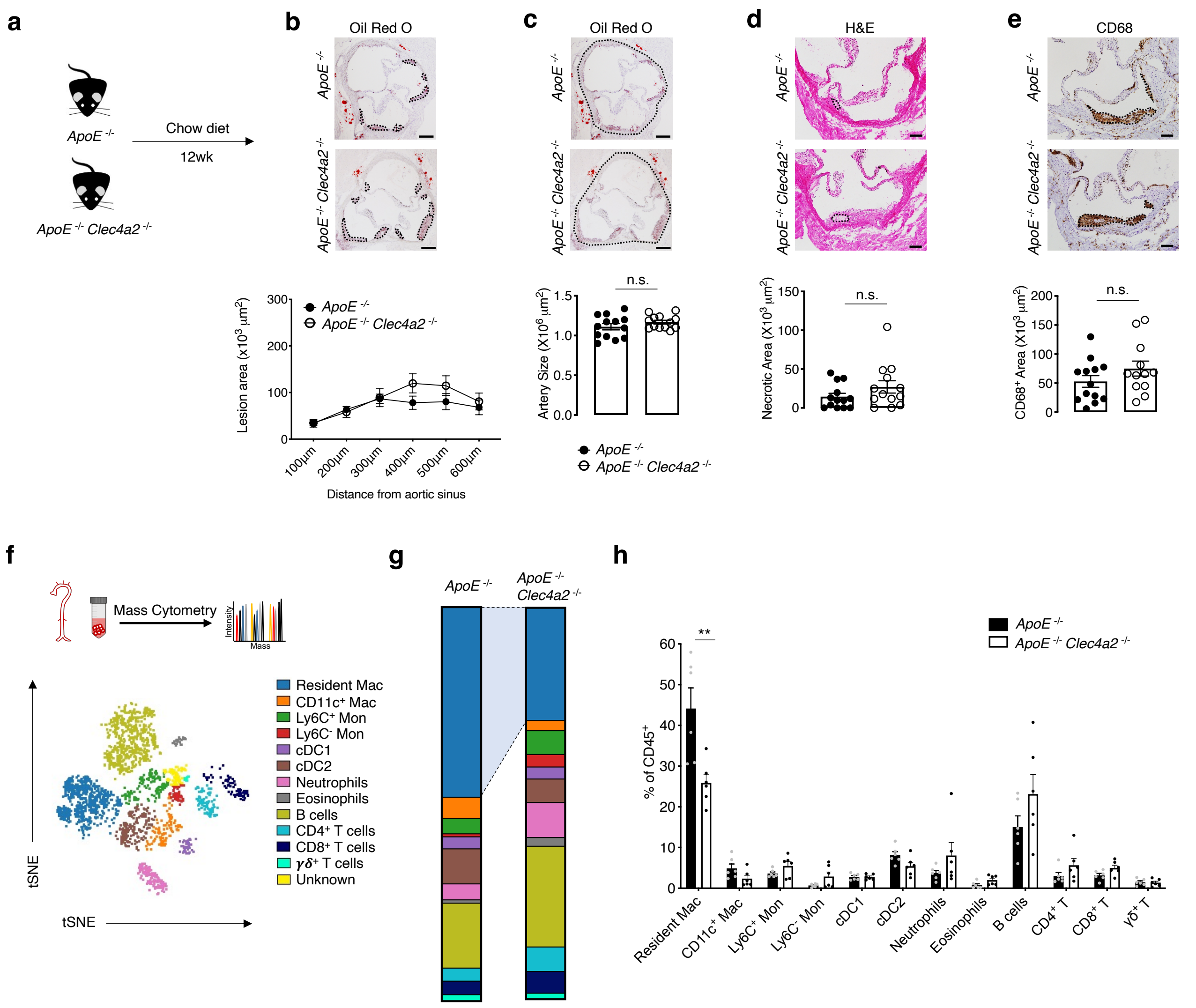

Supplementary Figure 7 (related to Figure 4).

a, Schematic showing experimental set up to compare 20-week-old *ApoE*-/- and *ApoE*-/- *Clec4a2*-/- littermate mice fed a chow diet for 12 weeks. **b,** Representative images and plaque quantification of Oil Red O-stained aortic roots of *ApoE*-/- and *ApoE*-/- *Clec4a2*-/- littermate mice (Scale bar: 200µm). n=13 mice each. Two-Way ANOVA. Not significant (n.s.). **c**, Representative images of aortic roots showing arterial size (dotted area) (Scale bar: 200*µ*m). n=13 mice each. Two tailed Student's t test. n.s. **d**, Representative images of aortic root lesions stained with haematoxylin and eosin (H&E) and the size of necrotic core (H&E-free area; dotted) (Scale bar: 100*µ*m). n=13 mice each. Two tailed Student's t test. n.s. **e,** Representative images and quantification of CD68-stained (brown)-area in aortic root lesions (dotted). (Scale bar: 100*µ*m). n=12-13 mice each. Two tailed Student's t test. n.s. **f,** tSNE clustering of live CD45+ cells from whole aortas of chow diet fed *ApoE*-/- and *ApoE*-/- *Clec4a2*-/- mice by mass cytometry. Leucocyte populations were identified based on expression of 32 markers. **g**, **h,** Frequency of aortic leucocyte populations in each genotype by mass cytometry. n=6; pooled from two independent experiments. Two tailed Student's t test. **P=0.0076. All data are presented as mean±SEM. Source data are provided as a Source Data file.

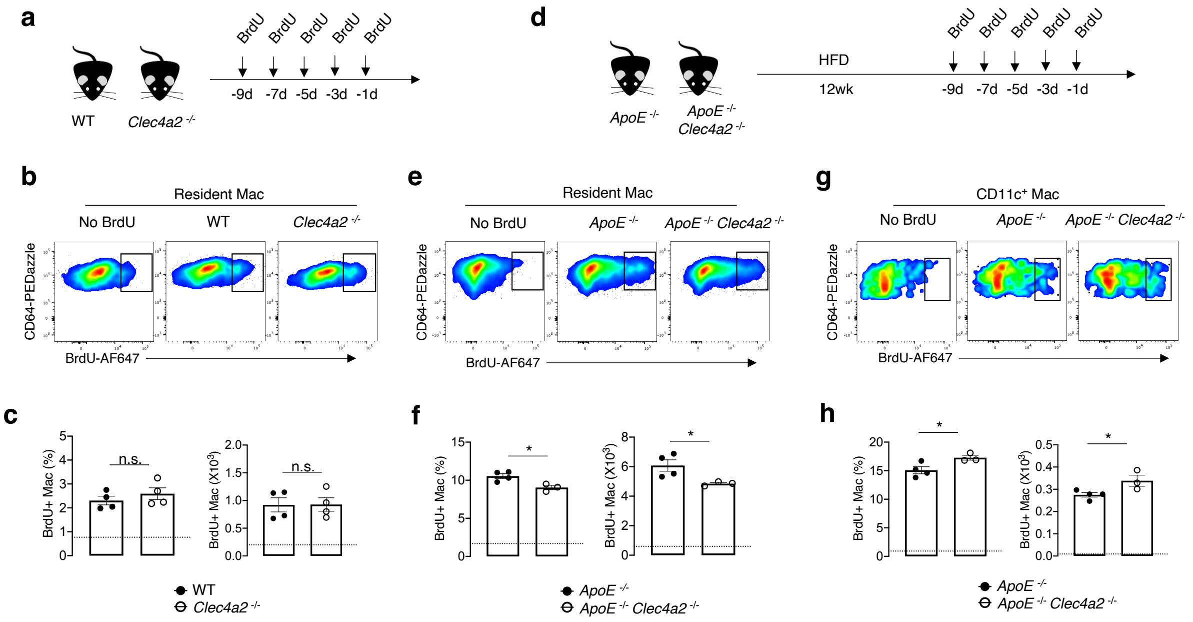

Supplementary Figure 8 (related to Figure 6).

a, Schematic of proliferation assays in WT and *Clec4a2*$^{-/-}$ mice. Bromodeoxyuridine (BrdU) was injected intraperitoneally every other day for 9 days. **b**, **c**, Representative images and percentages of BrdU incorporation in CD206^{+}Lyve1^{+} resident vascular macrophages. n=4 mice each; one experiment. Two tailed Student's t test. not significant (n.s.). **d**, Schematic of proliferation assays in *ApoE*$^{-/-}$ and *ApoE*$^{-/-}$ *Clec4a2*$^{-/-}$ mice fed an HFD for 12 weeks. BrdU was injected intraperitoneally every other day for 9 days before sacrifice. **e**, **f**, **g**, **h**, Representative images and percentages of BrdU incorporation in CD206^{+}Lyve1^{+} resident vascular macrophages and CD206^{-}Lyve1^{-}CD11c^{+} macrophages. n=3-4 mice each; one experiment. Two tailed Student's t test. Resident Mac: %: *P=0.0196, numbers: *P=0.0451, CD11c^{+} Mac: %: *P=0.0416, numbers: *P=0.0468. All data are presented as mean±SEM. Source data are provided as a Source Data file.

SUPPLEMENTARY TABLES

Supplementary Table 1. The effect of DT on immune cell populations in the heart, lungs, bone marrow and blood of *LysM*$^{Cre+}$ *Clec4a2*$^{flox/DTR}$ mice. The mice were subject to three diphtheria toxin (DT) or saline injections for a week. Cells from the heart and lungs were analysed using mass cytometry. Cells from the bone marrow (BM) and blood were examined using flow cytometry. n=4 mice. Two independent experiments. Data are presented as mean±SEM. Two-tailed Student's t-test. Source data are provided as a Source Data file.

Heart (Cell number X10^3)	Saline	DT	P values
Ly6C+ Mon	10.8±2.1	10.5±1.4	0.9822
cDC2	20.2±4.9	19.2±3.8	0.9822
Neutrophils	40.0±14.8	25.7±4.7	0.9170
cDC1	5.5±1.3	3.8±1.5	0.9170
CD4+ T cells	22.9±2.3	36.2±7.9	0.6439
CD8+ T cells	20.7±1.2	26.5±12.7	0.9620
B cells	224.4±6.1	156.3±34.4	0.5192
**Lung (Cell number X10^4)**	Saline	DT	P values
Interstitial Mac	0.6±0.1	0.1±0.1	0.1223
Ly6C+ Mon	4.1±0.8	15.4±2.4	0.0353
cDC2	1.1±0.2	4.9±1.4	0.1773
Neutrophils	9.0±1.5	15.6±1.9	0.1773
cDC1	1.5±0.2	3.70.6	0.1073
Eosinophils	7.2±2.2	6.7±1.7	0.9751
CD4+ T cells	9.7±1.5	14.4±1.6	0.2114
CD8+ T cells	6.0±0.9	9.5±0.9	0.1773
B cells	55.8±6.6	54.7±4.9	0.9751
**BM (Cell number X10^6)**	Saline	DT	P values
Neutrophils	6.2±0.8	4.2±1.7	0.3265
Ly6C+ Mon	1.7±0.2	2.5±0.3	0.0534
**Blood (%)**	Saline	DT	P values
Neutrophils	23.9±2.8	15.7±4.4	0.1629
Ly6C+ Mon	9.5±0.9	12.2±2.5	0.2900

Supplementary Table 2. Serum cholesterol and IL-6 levels in saline- or DT- treated *Ldlr*$^{-/-}$ chimera mice on high fat diet (related to Figure 3). n=7 mice. Data are presented as mean±SEM. Ordinary one-way ANOVA with Holm-Sidak's multiple comparisons test. Source data are provided as a Source Data file.

<table>
<tr><th>Host</th><th>Ldlr$^{-/-}$</th><th>Ldlr$^{-/-}$</th><th>Ldlr$^{-/-}$</th></tr>
<tr><td>Donor</td><td>LysM^{Cre+} Clec4a2$^{flox/DTR}$</td><td>LysM^{Cre+} Clec4a2$^{flox/DTR}$</td><td>Clec4a2$^{flox/DTR}$</td></tr>
<tr><td>Treatment</td><td>Saline</td><td>DT</td><td>DT</td></tr>
<tr><td>Cholesterol (mmol/L)</td><td>66.8±6.2</td><td>73.5±3.2</td><td>74.8±1.8</td></tr>
<tr><td>P values (saline vs DT)</td><td colspan="2">0.4713</td><td></td></tr>
<tr><td>P values (Control DT vs DT)</td><td></td><td colspan="2">0.8257</td></tr>
<tr><td>Serum IL-6 (ng/ml)</td><td>3.2±0.3</td><td>3.4±0.4</td><td>3.2±0.2</td></tr>
<tr><td>P values (saline vs DT)</td><td colspan="2">0.9491</td><td></td></tr>
<tr><td>P values (Control DT vs DT)</td><td></td><td colspan="2">0.9491</td></tr>
</table>

Supplementary Table 3. Percentages of immune cell populations in the aorta, lungs and bone marrow of WT and *Clec4a2*-deficient mice. Aorta and Lung: n=8 mice. BM: n=4 mice. Cells were analysed using flow cytometry. Data are presented as mean±SEM. Two-tailed Student's t-test. Source data are provided as a Source Data file.

**Aorta**	WT	*Clec4a2*$^{-/-}$	P values
Macrophages	40.4±4.2	39.0±5.3	0.9894
Ly6C^{+} Mon	1.3±0.3	1.6±0.5	0.9894
Neutrophils	3.0±0.6	2.8±0.4	0.9894
DCs	6.7±1.4	6.4±2.2	0.9894
**Lung**	WT	*Clec4a2*$^{-/-}$	P values
Alveolar Mac	9.15±1.4	8.7±1.7	0.9948
Interstitial Mac	0.8±0.1	0.6±0.1	0.5522
cDC1	1.2±0.1	1.4±0.1	0.9405
cDC2	0.8±0.1	1.0±0.1	0.8603
Ly6C^{+} Mon	4.6±0.6	4.6±0.6	0.9988
Ly6C^{-} Mon	3.1±0.2	3.1±0.4	0.9988
Neutrophils	7.6±0.3	8.2±0.5	0.9337
Eosinophils	3.5±0.3	3.3±0.2	0.9875
B cells	36.3±1.1	34.8±1.6	0.9769
T cells	23.3±1.6	24.4±2.0	0.9875
**BM**	WT	*Clec4a2*$^{-/-}$	P values
Macrophages	1.4±0.3	2.0±0.2	0.6491
Ly6C^{+} Mon	6.2±0.5	5.2±0.1	0.9422
Neutrophils	14.8±1.0	17.5±0.2	0.4747
Eosinophils	3.1±0.2	3.0±0.1	0.2743
HSCs	0.2±0.0	0.2±0.0	0.9544
CMPs	0.3±0.0	0.3±0.0	0.7307
GMPs	1.0±0.1	0.9±0.0	0.7210
MEPs	1.9±0.1	1.9±0.1	0.9544

Supplementary Table 4. Body weight and serum cholesterol levels of *ApoE*$^{-/-}$ and *ApoE*$^{-/-}$ *Clec4a2*$^{-/-}$ mice. The mice were fed a high fat diet (HFD) for 12 weeks. Body weight (Males n=16-22 mice, females n=14-17 mice). Serum cholesterol level (males n=10 mice). Data are presented as mean±SEM. Two-tailed Student's t-test. Source data are provided as a Source Data file.

	ApoE$^{-/-}$	*ApoE*$^{-/-}$ *Clec4a2*$^{-/-}$	P values
Male body weight (g)	34.4±0.9	34.4±0.8	0.9874
Female body weight (g)	23.3±0.3	22.8±0.3	0.2598
Cholesterol (mmol/L)	14.6±0.7	15.0±3.0	0.7380

Supplementary Table 5. Percentages of immune cell populations in the blood, bone marrow, spleen and para-aortic lymph nodes of *ApoE$^{-/-}$* and *ApoE$^{-/-}$ Clec4a2$^{-/-}$* mice. The mice were fed an HFD for 12 weeks. Blood: n=15 mice, BM: n=5 mice, spleen and PALN: n=10 mice. Cells from the spleen and PALN were analysed using mass cytometry. Cells from the BM and blood were examined using flow cytometry. Data are presented as mean±SEM. Two-tailed Student's t-test. Source data are provided as a Source Data file.

**Blood**	*ApoE$^{-/-}$*	*ApoE$^{-/-}$ Clec4a2$^{-/-}$*	P values
Neutrophils	21.6±4.4	12.8±2.9	0.1085
Eosinophils	2.1±0.4	1.3±0.4	0.1726
Ly6C^{+} Mon	**4.1±0.6**	**2.6±0.3**	**0.0355**
Ly6C^{-} Mon	4.5±1.0	3.5±0.7	0.4191
**BM**	*ApoE$^{-/-}$*	*ApoE$^{-/-}$ Clec4a2$^{-/-}$*	P values
Neutrophils	44.4±10.7	30.4±5.2	0.2720
Eosinophils	3.1±0.8	2.8±0.5	0.8010
Ly6C^{+} Mon	6.7±1.1	7.5±0.9	0.6050
HSCs	1.3±0.5	1.3±0.3	0.9191
CMPs	0.6±0.2	0.9±0.1	0.3103
GMPs	0.3±0.1	0.5±0.0	0.1195
MEPs	1.2±0.6	1.4±0.3	0.7039
**Spleen**	*ApoE$^{-/-}$*	*ApoE$^{-/-}$ Clec4a2$^{-/-}$*	P values
Total Mac	0.4±0.1	0.5±0.1	0.2104
Ly6C^{+} Mon	1.7±0.3	1.5±0.2	0.6732
Ly6C^{-} Mon	1.1±0.2	1.2±0.2	0.8704
cDC1	0.8±0.2	0.4±0.1	0.0568
cDC2	1.0±0.1	1.0±0.1	0.9853
Neutrophils	3.2±0.7	2.8±0.3	0.5916
Eosinophils	0.6±0.1	0.5±0.1	0.7682
B cells	50.2±1.8	51.0±1.8	0.7627
CD4^{+} T cells	20.7±1.3	20.5±1.0	0.8968
CD8^{+} T cells	10.6±0.9	10.0±0.4	0.5055
NK	2.9±0.3	2.8±0.3	0.8886
pDC	0.5±0.1	0.4±0.1	0.5413
**PALN**	*ApoE$^{-/-}$*	*ApoE$^{-/-}$ Clec4a2$^{-/-}$*	P values
DC/Mac	0.5±0.1	0.4±0.1	0.2545
B cells	44.0±4.2	48.6±5.9	0.5434
CD4^{+} T	26.2±2.2	24.5±2.8	0.6331
Ly6C^{-} CD8^{+} T	12.8±1.2	12.0±1.8	0.7161
Ly6C^{+} CD8^{+} T	11.2±0.9	9.0±0.7	0.0648
NK	0.7±0.1	0.8±1.2	0.6718

Supplementary Table 6. A list of differentially expressed genes (P<0.05) by CLEC4A2 deficiency that are found in both CSF1-BMDMs (*Clec4a2*$^{-/-}$ vs. WT mice) and vascular resident macrophages (Cluster 1; *ApoE*$^{-/-}$ *Clec4a2*$^{-/-}$ vs. *ApoE*$^{-/-}$ mice).

Downregulated	Upregulated
Ccr3	*Apobec1*
Prss23	*Mosmo*
Srp54a	*Il7r*
Bzw1	*Snx10*
Srp54c	*Snx15*
Clec4b1	*Jade2*
Tpbgl	*Depp1*
Tmem8	*Rgs1*
1700047I17Rik2	*Pi4k2b*
Clec4a4	*Cd1d1*
Cat	*4930503L19Rik*
Trim30a	*Mbd4*
Srp54b	*Plxnd1*
Clec4a2	*Rbp1*
Znf41-ps	*Vamp2*
Sema6d	*F10*
Zfp992	*Naip6*
Tmf1	
Ide	
Lpp	
Ids	
Cyp51	
G6pd2	
Ddx59	
Gosr2	
Atp6v1e1	
Fam177a	
Trib2	
Gpr183	
G530011O06Rik	
Gal	
Alox5	

Supplementary Table 7. A list of metal-conjugated antibodies for mass cytometry.

	Supplementary Figure 1a,b	Supplementary Figure 1f-i	Figure 2	Figure 4	Figure 6
141Pr	Ly-6G/C	Ly-6G/C	Ly-6G/C	Ly-6G/C	Ly-6G/C
142Nd	CD11c	CD11c	CD11c	CD11c	CD11c
143Nd	IL7ra	IL7ra	Tim4	IL-17ra	IL-17ra
144Nd	XCR1	XCR1	XCR1	XCR1	XCR1
145Nd	TCR$\gamma\delta$	TCR$\gamma\delta$	TCR$\gamma\delta$	TCR$\gamma\delta$	TCR$\gamma\delta$
146Nd	CX3CR1	CX3CR1	CX3CR1	CX3CR1	CX3CR1
147Sm	CD45	CD45	CD45	CD45	CD45.2
148Nd	CD11b	CD11b	CD11b	CD11b	CD11b
149Sm	CD19	CD19	CD19	CD19	CD19
150Nd	CD24	CD24	CD24	CD24	CD24
151Eu	CD64	CD64	CD64	CD64	CD64
152Sm	CD3e	CD3e	CD3e	CD3e	CD3e
153Eu	CD335	CD335	CD335	CD335	CD45.1
154Sm	CD31	CD43	-	CD103	CD43
155Gd	ACTA2	Lyve1	-	Lyve1	Lyve1
156Gd	CD169	CD169	CD169	CD43	CD169
158Gd	CD206	CD206	CD206	CD206	CD206
159Tb	F4/80	F4/80	F4/80	F4/80	F4/80
160Gd	CD26	CD26	CLEC4A2	CLEC4A2	CD26
161Dy	CD103	CD103	CD103	iNOS	CD103
162Dy	Ly6C	Ly6C	Ly6C	Ly6C	Ly-6C
163Dy	CCR2	CCR2	CCR2	CCR2	CCR2
164Dy	CD172a	CD172a	CD172a	CD172a	CD172a
165Ho	CD161	CD161	Lyve1	CD161	CD161
166Er	CD209b	CD209b	CD209b	CD209b	CD209b
167Er	SiglecF	SiglecF	SiglecF	SiglecF	SiglecF
168Er	CD8a	CD8a	CD8a	CD8a	CD8a
169Tm	TCRβ	TCRβ	TCRβ	TCRβ	TCRb
170Er	CD90.2	CD90.2	MerTK	CD90.2	CD90.2
171Yb	CD44	CD44	CD44	CD44	CD44
172Yb	CD4	CD4	CD4	CD4	CD4
173Yb	SiglecH	SiglecH	SiglecH	SiglecH	SiglecH
174Yb	MHCII	MHCII	MHCII	MHCII	MHCII
175Lu	CD68	CD68	CD68	CD68	CD68
176Yb	CD45R	CD45R	CD45R	CD45R	CD45R

Supplementary Table 8. Demographics of the human study population.

	Carotid
Number of subjects	29
Age. years (mean. SD)	69.3 (9.5)
Men (%)	62.1
Body mass index. kg/m^2 (median. SD)	25.6 (3.4)
History of smoking (%)	65.5
Diabetes (%)	13.8
Hypercholesterolemia (%)	75.9
Hypertension (%)	79.3
Statin user (%)	100.0
Blood pressure medication (%)	82.8
Diabetes medication (%)	3.4
Coronary artery disease (%)	34.5
Myocardial infarction (%)	13.8
Heart Failure (%)	13.8
