# Supplementary material for: C-type lectin receptor CLEC4A2 promotes tissue adaptation of macrophages and protects against atherosclerosis
Source: Nat Commun. 2022 Jan 11;13:215. doi: 10.1038/s41467-021-27862-9 (PMC8752790; doi:10.1038/s41467-021-27862-9)
Supplement: Supplementary file 3 — Description of Additional Supplementary Files [file 41467_2021_27862_MOESM3_ESM.pdf]

Description of Additional Supplementary Files

File name: Supplementary Data 1

Description: A list of differentially expressed genes (P<0.05) in 13 clusters of myeloid cells from the aortas of *ApoE*$^{-/-}$ mice by single cell RNA sequencing (related to Figure 1). Differentially expressed genes were identified using the two-sided Wilcoxon Rank Sum test with Bonferroni's correction for multiple hypothesis testing.

File name: Supplementary Data 2

Description: A list of inflammatory genes related to Supplementary Figure 2c.

File name: Supplementary Data 3

Description: A list of differentially expressed genes (P<0.05) in *Clec4a2*-deficient BMDMs relative to WT cells by RNA sequencing (related to Figure 5). Differential expression was assessed using the two-sided Robinson and Smyth exact test with Bonferroni's correction for multiple hypothesis testing.

File name: Supplementary Data 4

Description: A list of differentially expressed genes (P<0.05) in 13 clusters of myeloid cells from the aortas of *ApoE*$^{-/-}$ and *ApoE*$^{-/-}$ *Clec4a2*$^{-/-}$ littermate mice by single cell RNA sequencing (related to Figure 7). Differentially expressed genes were identified using the two-sided Wilcoxon Rank Sum test with Bonferroni's correction for multiple hypothesis testing.

File name: Supplementary Data 5

Description: A list of differentially expressed genes (P<0.05) in vascular resident macrophages (Cluster 1) of *ApoE*$^{-/-}$ *Clec4a2*$^{-/-}$ mice relative to *ApoE*$^{-/-}$ mice by single cell RNA sequencing (related to Figure 7). Differentially expressed genes were identified using the two-sided Wilcoxon Rank Sum test with Bonferroni's correction for multiple hypothesis testing.
