# Supplementary material for: C-type lectin receptor CLEC4A2 promotes tissue adaptation of macrophages and protects against atherosclerosis
Source: Nat Commun. 2022 Jan 11;13:215. doi: 10.1038/s41467-021-27862-9 (PMC8752790; doi:10.1038/s41467-021-27862-9)
Supplement: Supplementary file 9 — Reporting Summary [file 41467_2021_27862_MOESM9_ESM.pdf]

nature research

Corresponding author(s): Claudia Monaco

Last updated by author(s): 02-12-2021

Reporting Summary

Nature Research wishes to improve the reproducibility of the work that we publish. This form provides structure for consistency and transparency in reporting. For further information on Nature Research policies, see our Editorial Policies and the Editorial Policy Checklist.

Statistics

For all statistical analyses, confirm that the following items are present in the figure legend, table legend, main text, or Methods section.

n/a	Confirmed	
☐	☒	The exact sample size (*n*) for each experimental group/condition, given as a discrete number and unit of measurement
☐	☒	A statement on whether measurements were taken from distinct samples or whether the same sample was measured repeatedly
☐	☒	The statistical test(s) used AND whether they are one- or two-sided<br>*Only common tests should be described solely by name; describe more complex techniques in the Methods section.*
☐	☒	A description of all covariates tested
☐	☒	A description of any assumptions or corrections, such as tests of normality and adjustment for multiple comparisons
☐	☒	A full description of the statistical parameters including central tendency (e.g. means) or other basic estimates (e.g. regression coefficient) AND variation (e.g. standard deviation) or associated estimates of uncertainty (e.g. confidence intervals)
☐	☒	For null hypothesis testing, the test statistic (e.g. F, t, r) with confidence intervals, effect sizes, degrees of freedom and P value noted<br>*Give P values as exact values whenever suitable.*
☒	☐	For Bayesian analysis, information on the choice of priors and Markov chain Monte Carlo settings
☒	☐	For hierarchical and complex designs, identification of the appropriate level for tests and full reporting of outcomes
☒	☐	Estimates of effect sizes (e.g. Cohen's *d*, Pearson's *r*), indicating how they were calculated

Our web collection on statistics for biologists contains articles on many of the points above.

Software and code

Policy information about availability of computer code

Data collection	Data were collected using the following facilities: a Helios mass cytometer (Fluidigm), an LSRII (BD Biosciences) flow cytometer, an LSRFortessa (BD Biosciences) flow cytometer, a Chromium 10x Genomics platform, an Illumina HiSeq4000 platform for RNA sequencing and an Olympus BX51 osteometric brightfield and fluorescence microscope for imaging.
Data analysis	Software used for data analyses is outlined in Materials and Methods or Figure legends; these include FlowJo 10.5.3 for flow cytometry, Cytobank for mass cytometry, ivis algorithm for scRNA-seq (https://doi.org/10.1038/s41598-019-45301-0), ImageJ 2.1.0/1.53c (NIH) for imaging and Clemex Vision Lite 8.0.153 for image quantification. GraphPad Prism 9 was used for graphs and statistical analyses.

For manuscripts utilizing custom algorithms or software that are central to the research but not yet described in published literature, software must be made available to editors and reviewers. We strongly encourage code deposition in a community repository (e.g. GitHub). See the Nature Research guidelines for submitting code & software for further information.

Data

Policy information about availability of data

All manuscripts must include a data availability statement. This statement should provide the following information, where applicable:
- Accession codes, unique identifiers, or web links for publicly available datasets
- A list of figures that have associated raw data
- A description of any restrictions on data availability

The RNA-seq data are publicly available at ArrayExpress.

1) Fig1: Single cell RNA sequencing of myeloid cells from the aortas of ApoE-/- mice fed a high fat diet for 12-16 weeks.
Accession code: E-MTAB-10743 (https://www.ebi.ac.uk/arrayexpress/experiments/E-MTAB-10743/)

2) Fig5: Bulk RNA sequencing of CSF1-cultured bone marrow-derived macrophages from WT and Clec4a2-/- mice.
Accession code: E-MTAB-10734 (https://www.ebi.ac.uk/arrayexpress/experiments/E-MTAB-10734/)

3) Fig7: Single cell RNA sequencing of myeloid cells from the aortas of ApoE-/- and ApoE-/- Clec4a2-/- mice fed a high fat diet for 12 weeks.
Accession code: E-MTAB-10746 (https://www.ebi.ac.uk/arrayexpress/experiments/E-MTAB-10746/)

Field-specific reporting

Please select the one below that is the best fit for your research. If you are not sure, read the appropriate sections before making your selection.

☒ Life sciences ☐ Behavioural & social sciences ☐ Ecological, evolutionary & environmental sciences

For a reference copy of the document with all sections, see nature.com/documents/nr-reporting-summary-flat.pdf

Life sciences study design

All studies must disclose on these points even when the disclosure is negative.

Sample size	The minimum number of animals required were determined using power calculations with the G*Power software (85% power, standard deviation with an actual power (1-β error probability) of 0.85 and an α (error probability) of 0.05) to allow generation of meaningful data within the constraints of experimental variability.
Data exclusions	No exclusions were made.
Replication	Figures represent at least 2 independent experiments with multiple replicates (at least n>3 mice, the exact sample size is available in each Figure). Figure 1 includes 3 sets of scRNA-seq data which includes 9 mice per sample. In Figure 7a-c, each genotype group consists of 9 mice. The bulk RNA-seq data in Figure 5a,b include 3 individual mice per genotype. The mass cytometry data in Supplementary Figure 1a,b, Figure 4i-k and Supplementary Figure 7f-h were pooled from two to three experiments due to small cell number and small sample size. The independent experiment showed the similar results. Supplementary Figure 6, Figure 5f,h, Supplementary Figure 8 and Figure 7j,k represent one experiment with at least 3 biological replicates (mice).
Randomization	For all experiments, age/sex-matched mice were used to control for potential co-variates. Mice from the same cage were used when possible. When mice were purchased for experiments, they were maintained in the same room at least 2 weeks to allow acclimatisation.<br>For in vivo treatment experiments, mice from the same cage were randomly selected for different treatment. For genetic deletion experiments, littermate mice were selected based on their genotype. For in vitro experiments, cells from the same biological sample (mouse) were plated into separate wells as multiple replicates and the wells were randomly selected for different treatment.
Blinding	For histology quantification (lipid/CD68/CD64/CD11c/H&E), images were anonymously renamed to enable blind analysis. For other analyses, blinding was not required since experimental readouts were quantitative.

Reporting for specific materials, systems and methods

We require information from authors about some types of materials, experimental systems and methods used in many studies. Here, indicate whether each material, system or method listed is relevant to your study. If you are not sure if a list item applies to your research, read the appropriate section before selecting a response.

Materials & experimental systems

n/a	Involved in the study	
☐	☒	Antibodies
☒	☐	Eukaryotic cell lines
☒	☐	Palaeontology and archaeology
☐	☒	Animals and other organisms
☐	☒	Human research participants
☒	☐	Clinical data
☒	☐	Dual use research of concern

Methods

n/a	Involved in the study	
☒	☐	ChIP-seq
☐	☒	Flow cytometry
☒	☐	MRI-based neuroimaging

Antibodies

Antibodies used	Anti-mouse antibodies unless stated otherwise.<br><br>ACTA2 (conjugated with metal 155Gd in-house) Polyclonal Sigma-Aldrich Cat# A5228; 1:100<br>B220 (conjugated with metal 176Yb) RA3-6B2 Fluidigm Cat# 3176002B; 1:200

B220 (conjugated with PerCPCy5.5) RA3-6B2 Biolegend Cat# 103236; 1:200
Biotinylated Goat Anti-Hamster IgG N/A Vector Laboratories Cat# BA-9100; 1:200
Biotinylated Rabbit Anti-Rat IgG N/A Vector Laboratories Cat# BA-4001; 1:200
BrdU (conjugated with AF647) 3D4 BD Biosciences Cat# 560209; 1:50
CCR2 (conjugated with metal 163Dy in-house) QA18A56 Biolegend Cat# 357202; 1:100
CCR2 (conjugated with PE) QA18A56 Biolegend Cat# 150610; 1:100
CD103 (conjugated with metal 161Dy in-house) 2E7 Biolegend Cat# 121402; 1:100
CD11b (conjugated with BV711) QA19A45 Biolegend Cat# 101242; 1:200
CD11b (conjugated with FITC) QA19A45 Biolegend Cat# 101206; 1:200
CD11b (conjugated with metal 148Nd) QA19A45 Fluidigm Cat# 3148003B; 1:1000
CD11c N418 BioRad Cat# MCA1369GA; 1:100
CD11c (conjugated with APCCy7) N418 Biolegend Cat# 117324; 1:200
CD11c (conjugated with metal 142Nd) N418 Fluidigm Cat# 3142003B; 1:200
CD11c (conjugated with PE) N418 Biolegend Cat# 117308; 1:200
CD16/32 2.4G2 BD Biosciences Cat# 553142; 1:50
CD169 (conjugated with metal 156Gd in-house) 3D6.112 Biolegend Cat# 142402; 1:100
CD172a (conjugated with metal 164Dy in-house) P84 Biolegend Cat# 144002; 1:200
CD19 (conjugated with metal 149Sm) 6D5 Fluidigm Cat# 3149002B; 1:500
CD19 (conjugated with PerCPCy5.5) 6D5 Biolegend Cat# 152406; 1:200
CD206 MR5D3 BioRad Cat# MCA2235GA; 1:200
CD206 (conjugated with APC) C068C2 Biolegend Cat# 141708; 1:200
CD206 (conjugated with metal 158Gd in-house) C068C2 Biolegend Cat# 141702; 1:200
CD206 (conjugated with PECy7) C068C2 Biolegend Cat# 141720; 1:200
CD209b (conjugated with APC) 22D1 ThermoFisher Scientific Cat# 17-2093-82l; 1:50
CD209b (conjugated with metal 166Er in-house) 22D1 Biolegend Cat# 147802; 1:200
CD24 (conjugated with metal 150Nd) M1/69 Fluidigm Cat# 3150009B; 1:1000
CD26 (conjugated with metal 160Gd in-house) DPP-4 Biolegend Cat# 137802; 1:200
CD31 (conjugated with metal 154Sm in-house) 390 Abcam Cat# 102402; 1:200
CD3e (conjugated with metal 152Sm) 145-2C11 Fluidigm Cat# 3152004B; 1:200
CD3e (conjugated with PerCPCy5.5) 17A2 BD Biosciences Cat# 561108; 1:200
CD4 (conjugated with metal 172Yb) RM4-5 Fluidigm Cat# 3172003B; 1:500
CD43 (conjugated with metal 154Sm or 156Gd in-house) S11 Biolegend Cat# 143202; 1:100
CD44 (conjugated with metal 171Yb) IM7 Fluidigm Cat# 3171003B; 1:1000
CD45 (conjugated with BV785) 30-F11 Biolegend Cat# 103149; 1:200
CD45 (conjugated with FITC) 30-F11 Biolegend Cat# 103108; 1:200
CD45 (conjugated with metal 147Sm) 30-F11 Fluidigm Cat# 3147003B; 1:1000
CD45.1 (conjugated with PerCPCy5.5) A20 Biolegend Cat# 110728; 1:100
CD45.1 (conjugated with APC) A20 Biolegend Cat# 110714; 1:100
CD45.1 (conjugated with metal 153Eu) A20 Fluidigm Cat# 3153002B; 1:100
CD45.2 (conjugated with metal 147Sm) 104 Fluidigm Cat# 3147004B; 1:100
CD45.2 (conjugated with PE) 104 Biolegend Cat# 109808; 1:100
CD64 AT152-9 BioRad Cat# MCA5997; 1:100
CD64 (conjugated with APC) X54-5/7.1 Biolegend Cat# 305014; 1:200
CD64 (conjugated with metal 151Eu) X54-5/7.1 Fluidigm Cat# 3151012B; 1:200
CD64 (conjugated with PEDazzle) X54-5/7.1 Biolegend Cat# 139320; 1:200
CD68 FA-11 BioRad Cat# MCA1957GA; 1:200
CD68 (conjugated with metal 175Lu in-house) FA-11 Biolegend Cat# 137002; 1:200
CD8a (conjugated with metal 168Er) 53-6.7 Fluidigm Cat# 3168003B; 1:100
CD90.2 (conjugated with metal 170Er in-house) 30-H12 Biolegend Cat# 105302; 1:800
CX3CR1 (conjugated with metal 146Nd in-house) SA011F11 Biolegend Cat# 149002; 1:200
DCIR1/CLEC4A2 (biotinylated) TKKT-1; Professor Naoki Matsumoto (University of Tokyo); 1:500 (2ug/ml)
DCIR1/CLEC4A2 (conjugated with metal 169Gd in-house) TKKT-1; Professor Naoki Matsumoto (University of Tokyo); 1:200
F4/80 (conjugated with BV605) BM8 Biolegend Cat# 123133; 1:50
F4/80 (conjugated with metal 159Tb) BM8 Fluidigm Cat# 3159009B; 1:100
Goat-Anti-Hamster IgG-AF568 N/A ThermoFisher Scientific Cat# A-21112; 1:200
Goat-Anti-Rat IgG-AF488 N/A ThermoFisher Scientific Cat# A-11006; 1:200
Goat-Anti-Rat IgG-AF568 N/A ThermoFisher Scientific Cat# A-11077; 1:200
IL-7Ra (conjugated with metal 143Nd in-house) A7R34 Biolegend Cat# 135002; 1:100
iNOS (conjugated with metal 161Dy) CXNFT Fluidigm Cat# 3161011B; 1: 100
Ly6C (conjugated with metal 162Dy) HK1.4 Fluidigm Cat# 3162014B; 1:1000
Ly6C (conjugated with PECy7) HK1.4 Biolegend Cat# 128018; 1:200
Ly6G (conjugated with PerCPCy5.5) 1A8 Biolegend Cat# 127616; 1:200
Ly6G (conjugated with PECy7) 1A8 Biolegend Cat# 127618; 1:200
Ly6G/C (conjugated with metal 141Pr) RB6-8C5 Fluidigm Cat# 3141005B; 1: 1000
Lyve1 ALY7 ThermoFisher Scientific Cat# 14-0443-82; 1:200
Lyve1 (conjugated with metal 155Gd in-house) ALY7 ThermoFisher Scientific Cat# 14-0443-82; 1:200
Lyve1 (conjugated with metal 162Ho in-house) ALY7 ThermoFisher Scientific Cat# 14-0443-82; 1:200
MerTK (conjugated with metal 170Er in-house) Polyclonal R&D Systems Cat# AF591; 1:100

MHCII (conjugated with BV421) M5/114.15.2 BD Biosciences Cat# 562564; 1:200
MHCII (conjugated with metal 174Yb) M5/114.15.2 Fluidigm Cat# 3174003B; 1:1000
NK1.1 (conjugated with metal 165Ho) PK136 Fluidigm Cat# 3165018B; 1:100
NKp46 (conjugated with metal 153Eu) 29A1.4 Fluidigm Cat# 3153006B; 1:100
SiglecF (conjugated with metal 167Er in-house) E50-2440 BD Biosciences Cat# 552125; 1:100
SiglecF (conjugated with PE) E50-2440 BD Biosciences Cat# 562068; 1:200
SiglecF (conjugated with PerCPCy5.5) E50-2440 BD Biosciences Cat# 565526; 1:200
SiglecH (conjugated with metal 173Yb in-house) 551 Biolegend Cat# 129602; 1:100
TCRb (conjugated with metal 169Tm) H57-597 Fluidigm Cat# 3169002B; 1:500
TCRgd (conjugated with metal 145Nd in-house) GL3 Biolegend Cat# 118101; 1:100
Tim4 (conjugated with metal 143Nd in-house) RMT4-54 Biolegend Cat# 130002; 1:100
XCR1 (conjugated with metal 144Nd in-house) ZET Biolegend Cat# 148202; 1:200

Validation

Commercially available antibodies were validated by the manufacturers; anti-mouse antibodies were validated by using mouse tissues (bone marrow cells, peritoneal macrophages or splenocytes) or mouse cell lines (J774A.1). Some antibodies for mass cytometry conjugated with metal in-house were validated and optimised using murine aortic, bone marrow or splenic cells. Purified antibodies for histology were optimised using mouse tissue sections prior to use for experiments.

Animals and other organisms

Policy information about studies involving animals; ARRIVE guidelines recommended for reporting animal research

Laboratory animals

Mouse strains used for experiments were matched by age and sex. Both males and females were used and were equally divided into each experimental group. For RNA sequencing (Fig1, Fig5 and Fig7) and in vivo proliferation assays (Supplementary Figure 8), female mice were used. For atherosclerosis research, 8-10-week-old ApoE-/- mice received a HFD or remained on a chow diet and were sacrificed at the age of 20-26-week. 8-10-week-old Ldlr-/- mice underwent irradiation and bone marrow transfer and were sacrificed at the age of 24-30-week. For all other experiments, mice at 8-12-week-old were used.

ApoE-/- Charles River Laboratories UK CR: 622 or JAX: 002052
ApoE-/- Clec4a2-/- Bred in-house
LysMCre Jackson Laboratory US JAX: 004781
Ldlr-/- Jackson Laboratory US JAX: 002207
CD45.1 Envigo UK JAX: 002014
C57BL/6J Charles River Laboratories UK CR: 632 or JAX: 000664
Cx3cr1eGFP Jackson Laboratory US JAX: 005582
CD45.1 CD45.2 heterozygous Bred in-house
Clec4a2-/- Fujikado et al., 2008 (DOI: 10.1038/nm1697)
Clec4a2flox/DTR Gen0way N/A
Cx3cr1eGFP CD45.1 CD45.2 heterozygous Bred in-house
Ldlr-/- CD45.1 Bred in-house
LysMCre Clec4a2flox/DTR Bred in-house

Wild animals

No wild animals were used in this study.

Field-collected samples

No field-collected samples were used in this study.

Ethics oversight

All experimental animal procedures were approved by The University of Oxford Animal Welfare Ethical Review Board and performed according to UK Home Office regulations and The University of Oxford guidelines. UK Home office regulations conform to the guidelines from Directive 2010/63/EU of the European Parliament on the protection of animals used for scientific purposes.

Note that full information on the approval of the study protocol must also be provided in the manuscript.

Human research participants

Policy information about studies involving human research participants

Population characteristics

The mean age of the patients was 69.3±9.5 years (mean±SD) and 62.1% of the participants were male (n=29). All patients received statins; 82.8% of them received blood pressure medication; 3.4% of them received diabetes medication. The clinical characteristics are reported in the Supplementary Table 8.

Recruitment

Vascular sample series from the Tampere Vascular Study (TVS) were obtained during open vascular procedures from patients fulfilling the following inclusion criteria: carotid endarterectomy because of asymptomatic or symptomatic and hemodynamically significant (>70%) carotid stenosis. Exclusion criterion was a lack of patient's consent to participate in the study. All studies were conducted according to the declaration of Helsinki, and the study subjects gave informed consent. The samples were taken from patients subjected to open vascular surgical procedures in the Division of Vascular Surgery and Heart Center, Tampere University Hospital. Patients were prospectively enrolled from surgical revascularization lists at any

	given day to exclude any potential bias.
Ethics oversight	The Tampere Vascular study was approved by the Ethics Committee of Pirkanmaa Hospital District (Finland).

Note that full information on the approval of the study protocol must also be provided in the manuscript.

Flow Cytometry

Plots

Confirm that:

- [x] The axis labels state the marker and fluorochrome used (e.g. CD4-FITC).
- [x] The axis scales are clearly visible. Include numbers along axes only for bottom left plot of group (a 'group' is an analysis of identical markers).
- [x] All plots are contour plots with outliers or pseudocolor plots.
- [x] A numerical value for number of cells or percentage (with statistics) is provided.

Methodology

Sample preparation	Tissues were cut into small pieces and digested in the appropriate enzyme mixture at 37 °C in a water bath shaker: Aorta: 450 U/ml collagenase I, 125 U/ml collagenase XI 60 U/ml hyaluronidase and 60 U/ml DNase I for 50 minutes; Lung: 0.4 mg/ml collagenase IV and 0.15 mg/ml DNase I for 40 minutes; Heart: 1 mg/ml collagenase II and 0.15 mg/ml DNase I for 30 minutes. The cells were retrieved by passing tissue pieces through a 70 µm cell strainer. Spleen and lymph nodes were mechanically dissociated by passing cells through a 70µm cell strainer. For separating aortic layers into the intima/media and the adventitia, the whole aorta was pre-incubated with 125 U/ml collagenase II and 3.75 U/ml elastase at 37 °C for 10 minutes. The adventitia layer was separated from the inner tube (the intima and media) by pulling with two pairs of fine forceps. The tissues were then digested as performed in the aorta sample. For lungs, hearts and spleens, erythrocytes were lysed using Red Cell Lysis Buffer at room temperature for 2 minutes.<br><br>Non-specific binding of antibodies was blocked by incubating cells with rat anti-mouse CD16/32 at 4 °C for 10 minutes, followed by incubating with fluorochrome-conjugated antibodies recognising cell surface markers for 30 minutes at 4 °C. Following by the fixation step, cell acquisition was performed using a flow cytometer.
Instrument	For data acquisition, LSRII (BD Biosciences) or LSRFortessa (BD Biosciences) flow cytometer was used. For sorting, FACSAria III (BD Biosciences) was used.
Software	Data were analysed using FlowJo software .
Cell population abundance	The purity of the sorted cells was confirmed immediately after sorting and it was over 98%.
Gating strategy	Overall, cells were gated by FSC/SSC to remove debris. Only single cells were selected using FSC-A/FSC-W and SSC-A/SSC-H. Dead cells were removed using a Live/Dead fixable aqua dye, and live CD45+ cells were further gated for population of interest based on marker expression. Gating strategy is available in Figures, Supplementary Figures or Figure legends.

- [x] Tick this box to confirm that a figure exemplifying the gating strategy is provided in the Supplementary Information.
